# 

**Published:** 1917-06-02

**Authors:** 


### CONTENTS.

PAGE

**The General Medical Council and the Supply of Doctors** ... 161

**The Medical Profession and the Uncertified Midwife** ... 162

**Hospital and Institutional News** ... 163
American Medical Units for the Front—An Institutional Law-Suit—The Anglo-Russian Hospital at Petrograd—The Earl of Lichfield's Opportunity—The Most Christian of Institutions—A Nottinghamshire "Star and Garter"—The Prince of Wales' Hospital for Limbless Sailors and Soldiers, Cardiff—Hospital Collector Killed by the Germans—R.A.M.C. Nomenclature—East End Sportsmen—A New Hospital for Bristol—A Valuable Extension—The Absent Ambulance—A New Form of Economy—Cash or Credit?—Cottage Hospitals and the War—The Roll of Honour Hospital—The Treatment of Neurasthenics—An Excellent Story—The Cares of the Caterer—The Food Controller and Milk Contracts—Institutional Staffs and Reduced Food—Oscar Wilde and Hospital Appeals—A Fall in Infant Mortality—An Editor on Her Merits—Legacies for Plymouth Institutions—This Week's Drug Market.

**Medical Organisation** ... 169
The Fabian Report on Professional Associations.

**Occupational Therapy** ... 170

**The Institutional Library** ... 171
Science and the Nation—Report of the Radium Institute—Reviews in Brief.

**Parliament and the Profession** ... 173

**Memorandum by the Council on the Petition to the Prime Minister** ... 174

**The Matrons' and Sisters' Department** ... 175
The Preliminary Training School for Nurses.
Round the Hospitals.
Suffragettes at Queen's Hall—College Meeting at Norwich—Edith Cavell Memorial—Standardising Nurse-Training Records—The Supply of Nurses.

**The Editor's Letter-Box** ... vi

Clayton Hospital and Wakefield General Dispensary.

**Mothers' Pensions** ... vi

**The Institutional Worker** ... (Suppl.) 1
Electro-Medical Apparatus: Their Use, Construction, and Care.
Question for June.
Questions Invited.
Enquiries and Answers.
Economy in Repairing Surgeons' Rubber Gloves.

